# SOX2 and cancer: current research and its implications in the clinic

**DOI:** 10.1186/2001-1326-3-19

**Published:** 2014-07-04

**Authors:** Kasia Weina, Jochen Utikal

**Affiliations:** 1 Skin Cancer Unit, German Cancer Research Center (DKFZ), Heidelberg, Germany; 2 Department of Dermatology, Venereology and Allergology, University Medical Center Mannheim, Ruprecht-Karls-Universität Heidelberg, Theodor-Kutzer-Ufer 1-3, 68135 Mannheim, Germany

**Keywords:** *SOX2*, Stem cell marker, Reprogramming, Cancer, Cancer stem cells, Biomarker, Prognosis

## Abstract

*SOX2* is a gene that encodes for a transcription factor belonging to the *SOX* gene family and contains a high-mobility group (HMG) domain, which permits highly specific DNA binding. Consequently, SOX2 functions as an activator or suppressor of gene transcription. *SOX2* has been described as an essential embryonic stem cell gene and moreover, a necessary factor for induced cellular reprogramming. *SOX2* research has only recently switched focus from embryogenesis and development to SOX2’s function in disease. Particularly, the role of *SOX2* in cancer pathogenesis has become of interest in the field. To date, studies have shown SOX2 to be amplified in various cancer types and affect cancer cell physiology *via* involvement in complicated cell signaling and protein-protein interactions. Recent reviews in this field have highlighted SOX2 in mammalian physiology, development and pathology. In this review, we comprehensively compile what is known to date about SOX2’s involvement in cancer biology, focusing on the most recent findings in the fields of cellular signaling and cancer stem cells. Lastly, we underscore the role of SOX2 in the clinic and highlight new findings, which may provide novel clinical applications for SOX2 as a prognostic marker, indicator of metastasis, biomarker or potential therapeutic target in some cancer types.

## Introduction

The SOX family is a group of related transcription factors that have demonstrated their importance in developmental and stem cell biology. In 1990, pioneering research discovered the mammalian testis-determining factor and the gene was termed *Sry* due to its corresponding location in the sex-determining region on the Y-chromosome [1,2]. *Sry* contains a distinctive high-mobility group (HMG) domain, which permits precise DNA recognition and binding. Proteins that contain the HMG domain with amino acid similarity of 50% or higher to the HMG domain of *Sry* are termed SOX (abbreviation for Sry-related HMG box) proteins [3-5]. Known functions of these proteins range from regulation of embryonic development and stem cell maintenance to homeostasis in adult tissues [6].

**Figure 1:**
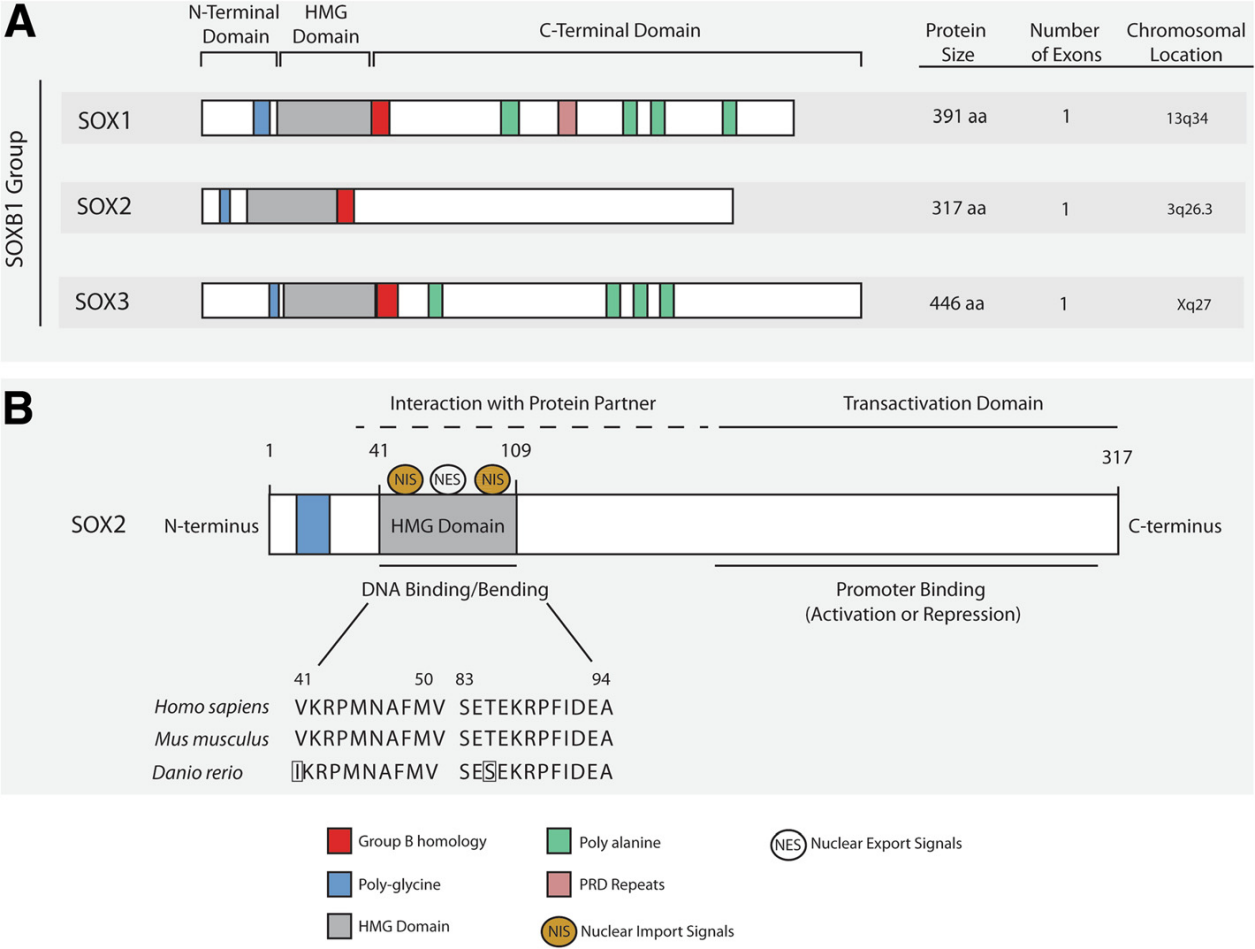
**SOX2 homology, structure and protein function. (A)** SOX2 belongs to the SOXB1 of SOX proteins. There is large homology between the SOXB1 group and they all contain three major domains: N-terminal, HMG and C-terminal domain. **(B)** SOX2 protein domains play several functional roles. The HMG domain of SOX2 remains fairly conserved between *homo sapiens*, *Mus musculus* and *Danio rerio* (Swiss-Prot: P48431, P48432, Q6P0E1). The HMG domain also serves as potential binding sites for protein partners. Moreover, nuclear import signals (NIS) and nuclear export signals (NES) bind to the HMG domain regulating SOX2 itself. Lastly the transactivation domain functions as the region responsible for promoter binding, which in turn leads to activation or repression of target genes.

In 1994, the *SOX2* gene, one of the *SOX* family members, was discovered and characterized in humans [7]. The *SOX2* gene is located on chromosome 3q26.3–q27, belongs to the SOXB1 group and encodes for a protein consisting of 317 amino acids (Figure 1A) [7,8]. SOX2 is comprised of three main domains: N-terminal, HMG and transactivation domain (Figure 1B). SOX2 research thus far has heavily emphasized its crucial role in stem cell maintenance, lineage fate determinant and a necessary factor to reprogram somatic cells back towards pluripotency [5,9,10]. In disease, *SOX2* alterations have been associated with developmental maladies, such as anophthalmia-esophageal-genital (AEG) syndrome, which occurs when there is a heterozygous mutation of *SOX2* that leads to abnormal development of ectodermal and endodermal tissues [11]. Aside from developmental diseases, accruing research has strongly associated SOX2 with cancer.

Here, we consolidate SOX2’s role in cancer and provide a comprehensive overview of the field, focusing on the latest research that has implicated SOX2 in cancer biology and in the clinic. SOX2 has been shown to be associated with numerous cancer types, even described in some cases as an oncogene, and controls cancer cell physiology *via* promoting oncogenic signaling and maintaining cancer stem cells. Lastly, we investigate recent work that has highlighted the role of *SOX2* in the clinic, particularly its influence on prognosis, therapy resistance and potential therapeutic interventions.

## Review

### SOX2 and cancer biology: current research

#### SOX2 amplification in cancer

Tumor development occurs when a cell accumulates genetic alterations that modify normal cell cycle progression. There are several means of genetic aberrations including gene deletions or gene amplification. Gene amplification is defined as a copy number increase of a particular chromosomal region. *SOX2* amplification is due to multiplication of the 3q26.3 gene locus [12,13]. *SOX2* amplification has previously been found in several cancer types including glioblastoma, small-cell lung cancer (SCLC) and many forms of squamous cell carcinoma (SCC) [12,14-20]. A summary of SOX2 amplification in various cancer types with corresponding references can be found in Table 1.

**Table 1:** Summary of SOX2 amplification and functions in cancer

**Cancer type**	***SOX2 *** **gene amplification**	**SOX2 function**	**Pathway/Process**	**References**
Breast	No	↑cell proliferation, ↑colony formation, ↑↓invasion, ↑metastasis	↑WNT/ β-CATENIN, ↑EMT, ↓AMPK/mTOR	[21-27]
Cervical	Unknown	↑cell proliferation, ↑clonogenicity, ↑tumorigenicity	Unknown	[28]
Colorectal	Unknown	↑↓cell proliferation, ↑metastasis, ↑senescence, ↑autophagy, ↑tumor growth, ↑invasion, ↑migration, ↑anchorage-dependent growth	↑BMP, ↓mTOR,↑MET, ↑WNT/ β-CATENIN	[29-33]
Esophageal SCC	Yes	↑cell proliferation, ↑tumor growth	↑Akt/mTORC1, ↑STAT3	[17,34-36]
Gastric	Unknown	↑↓ apoptosis, ↑↓cell proliferation, ↑migration	↑AKT signaling	[37-41]
Glioblastoma, GBM, medulloblastoma, oligodendroglioma	Yes	↑promoter hypomethylation, ↑invasion, ↑migration, ↑self-renewal CSCs, ↑cell proliferation, ↑colony formation	Unknown	[14,15,42-46]
Hepatocellular carcinoma	Unknown	↑invasion, ↑sphere formation	↑EMT	[47]
Layngeal	Unknown	↑ invasion/migration	↑MMP-2 ↑PI3L/AKT/mTOR	[48]
Melanoma	Unknown	↑invasion, ↑tumor volume, ↑self-renewal CSCs	↑Hedegehog-GLI signaling	[49-53]
Oral SCC	Yes	Unknown	Unknown	[20]
Osteosarcoma	Unknown	↑self-renewal CSCs, ↑tumorigenicity, ↑dedifferentiation	↓WNT/ β-CATENIN	[54]
Ovarian	Unknown	↑migration, ↑invasion, ↑colony formation	Unknown	[55,56]
Pancreatic	Unknown	↑cell proliferation, ↑stemness/dedifferentiation	↑EMT	[57]
Prostate	Unknown	↑self-renewal CSCs, ↑cell proliferation, ↑cell survival, ↑metastasis, ↑migration, ↓apoptosis, ↓store-operated Ca^2+^ entry	↓Ca^2+^ channels, ↑EMT, ↓Survivin, ↑WNT/β-CATENIN, ↑EGFR/PI3K/AKT	[27,58-60]
SCLC, Lung SCC, lung adenocarcinoma, NSCLC	Yes	↑cell proliferation, ↑cell survival, ↓apoptosis, ↑migration, ↑anchorage-dependent growth, ↑self-renewal CSCs, ↑metastasis, ↓autophagy, ↑tumor formation	↑MAP4K4-Survivin, ↓EGFR/Src/Akt ↓BMP4	[12,16,19,61-70]
Sinonasal	Yes	Unknown	Unknown	[71]
Transitional cell Carcinoma	Unknown	↑alternative splicing	Unknown	[72]

SOX2 has been shown to be amplified and functionally relevant in various cancer types. SOX2 in cancer functions through multiple mechanisms that vary depending on the cancer type. However, in most cases, SOX2 has been shown to increase cell proliferation, invasion, migration, metastasis and self-renewal of CSCs. In addition, some of these phenotypes have been linked to particular oncogenic pathways, including WNT/β-CATENIN, EGFR, mTOR and HH signaling.

↑ = Promotes/Improves.

↓ = Suppresses/Inhibits.

↑↓ = Conflicting research.

The latest *SOX2* studies have focused on the co-amplification of *SOX2* with other critical genes. Justilien and colleagues revealed the co-amplification of two oncogenes, *PRKCI* and *SOX2*, is responsible for the cancer stem cell phenotype seen in lung squamous cell carcinoma (LSCC) [61]. Moreover, another study performed FISH analysis in 447 resected non-small cell lung cancer (NSCLC) tissue samples and *SOX2* amplification was associated with increased gene copy number of *FGFR1* and *PI3KCA* genes [62].

#### SOX2’s involvement in cancer cell physiology varies between cancer types

**Figure 2:**
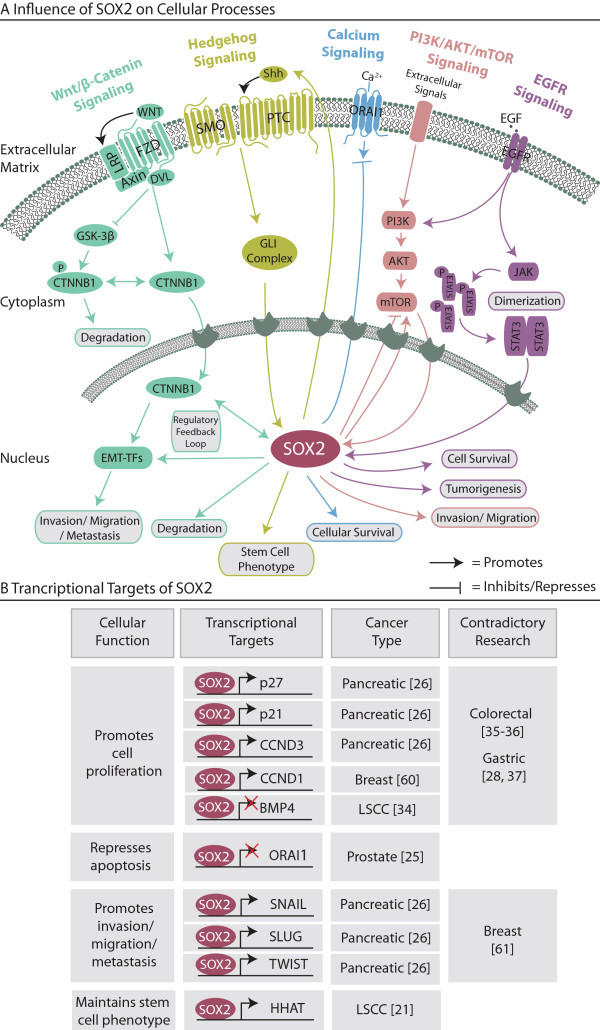
**Influence of SOX2 on oncogenic-related processes and transcription. (A)** SOX2 is an important regulator of cellular processes related to cancer. Some of these processes include but aren’t limited to WNT/β-CATENIN signaling, EMT and JAK/STAT3 signaling. In most cases, SOX2 functions downstream in the nucleus. SOX2’s activity leads to further downstream effects and finally alters cellular phenotypes such as cellular survival, invasion and metastasis. **(B)** SOX2 is typically regulating processes downstream on a transcriptional level. There are several examples of SOX2 influencing cancer phenotypes by repressing or activating particular target genes including EMT promotion *via* binding to the promoter regions of *SNAIL*, *SLUG* and *TWIST*. Therefore, SOX2 functions in cancer as a key transcription factor.

As described by Hanahan and Weinberg, cancer is a disease characterized by determined hallmarks some of which are: sustained proliferative signaling, activation of invasion and metastasis, and evasion of cell death [73]. Studies have strongly associated SOX2 to these respective cancer hallmarks and thus far SOX2 has been shown to promote cellular proliferation (breast, prostate, pancreatic and cervical cancers) [21,28,57,58], evade apoptotic signals (prostate, gastric cancer and NSCLC) [37,58,63] and promote invasion, migration and metastasis (melanoma, colorectal, glioma, gastric, ovarian cancer and hepatocellular carcinoma) [15,29,47,49,55]. We summarized *SOX2* amplification and resulting alterations in cellular functions in all cancer types in Table 1 and showed examples of SOX2’s role in oncogenic signaling in Figure 2. Below we highlight a few functional examples of SOX2 in cancer before we review the latest SOX2 research in different aspects of cancer physiology including: cellular proliferation, apoptosis and invasion/migration/metastasis. For a complementary and closer examination into SOX2 and oncogenic signaling, protein-protein interactions and miRNAs see review by Liu *et al.*[74].

Cellular proliferation is tightly regulated by SOX2 in many cancer types. SOX2 knockdown in pancreatic cancer cells resulted in cell growth inhibition through cell cycle arrest, not apoptosis, *via* the transcriptional induction of p21^Cip1^ and p27^Kip1^[57]. When SOX2 was overexpressed, cell proliferation was promoted through cyclinD3 (CCND3) transcriptional induction and allowed further S-phase entry (Figure 2B) [57]. Recently, Hütz and colleagues confirmed this in gastric cancer [37]. SOX2 was functionally inhibited *via* cellular transfection with a tetracycline-inducible C-terminally truncated version of SOX2, termed dnSOX2. Despite lacking the transactivation domain, dnSOX2 could bind SOX2 recognition sites on DNA and compete with wild-type SOX2. This inhibition led to the decrease in cellular proliferation in AZ-521 cells and further analysis using a RNA gene expression microarray, revealed an upregulation of p21 and downregulation of Δp63 (splice variant of p63) [37]. Additionally, Fang and colleagues found in LSCC, SOX2-silencing inhibited cellular proliferation *via* the upregulation of BMP4 [64]. After performing chromatin immunoprecipitation and luciferase experiments, SOX2 was found to transcriptionally repress the *BMP4* promoter. The authors therefore suggest that *BMP4* is playing a tumor suppressor role in LSCC, while SOX2 repression of *BMP4* transcription causes cell growth [64]. It’s important to note that the involvement of SOX2 in cell proliferation has been controversially discussed in colorectal and gastric cancer [30,31,37,38]. The contradictory effect of SOX2 in cell proliferation suggests that SOX2 plays a differential role depending on the type of cancer (Figure 2B).

SOX2 also plays an important role in evading apoptotic signals. In prostate cancer, *in vitro* and *in vivo* xenograft experiments using DU145 SOX2-overexpressing cells in NOD/SCID mice revealed that SOX2 caused an increase in apoptotic resistance by decreasing store-operated Ca^2+^ entry *via* repressing *ORAI1* expression (Figure 2) [58]. Equivalently, upon silencing of *SOX2* in NSCLC cell lines, apoptosis was induced [63]. Hütz and colleagues, in addition to proliferation analysis, investigated apoptosis using, caspase 3/7 assays performed in AZ-521 gastric cancer cells [37]. After 48 hours of SOX2 inhibition, nearly 60% of cells were apoptotic compared to only 20 – 40% of the control cells [37]. These studies prove SOX2’s involvement in apoptosis inhibition and therefore in direct promotion of uncontrolled cell growth.

Finally, research has indicated that SOX2 is a novel regulator of cell invasion, migration and metastasis. For example in melanoma, SOX2 knockdown in A2058 cells resulted in a 4.5-fold decrease in invasion *in vitro* and adopted this phenotype *via* the upregulation of matrix metalloproteinase (MMP)-3 [49]. Likewise in colorectal cells, SOX2 was involved in cellular migration and invasion *in vitro,* but mediated these effects through MMP-2 [29]. This invasive phenotype was also confirmed in malignant glioma, since siRNA-mediated downregulation of SOX2 resulted in a significant decrease in migration and invasion capabilities [15]. Moreover SOX2 overexpression in the SOX2-negative glioma cell line U-87 resulted in a significant increase in the number of migratory and invasive cells [15]. Numerous gain and loss of function studies in several cancer types (gastric cancer, ovarian cancer and hepatocellular carcinoma) reinforced the link between SOX2 and cellular invasion and migration [15,37,47,55]. Recently, Yang and colleagues showed SOX2’s involvement in promoting invasion and migration in laryngeal cancer cells through the induction of MMP-2 and the PI3K/AKT/mTOR pathway (Figure 2A) [48].

#### SOX2 and cancer stem cells: current research

Many tumors are derived from a single cell that has undergone malignant transformation through the acquisition of genomic aberrations, e.g. gene amplification, mutations or other mechanisms [75]. Clonal expansion of cells with tumorigenic properties is the next step towards tumor initiation. These selected cells have the ability to evade normal cell cycle checkpoints, rapidly proliferate and invade tissues [73,75]. The orchestration of tumor initiation and maintenance has been shown in some cancers to be driven by cancer stem cells (CSCs), also termed tumor-initiating cells or cancer stem-like cells. These CSCs may acquire tumor-initiating and self-renewal properties through similar molecular mechanisms governing cellular reprogramming [76]. Evidence has linked induced cellular reprogramming to cancer and led to the assumption that CSCs may arise *via* a reprogramming-related mechanism [77-79]. The reactivation of stem cell-associated markers or pluripotency factors may cause dedifferentiation and a more stem cell-like state [76]. Sussman and colleagues discovered that the ubiquitin-specific protease 22 (USP22) is responsible for controlling the cellular transition from stemness towards differentiation [80]. Moreover they found USP22 represses the *SOX2* promoter in order to control the embryonic stem cell transition from self-renewal to differentiation [80] Therefore, not only is *SOX2* an essential stem cell marker but its suppression is mandatory for cellular differentiation. For these reasons, SOX2 has been heavily investigated in CSCs in several cancer types.

#### SOX2 regulates self-renewal and maintenance in cancer stem cell populations

*SOX2* has shown to increase CSC markers in ovarian, pancreatic, lung cancer, but research also has proven its function in self-renewal [57,81,82]. Self-renewal capacity of a CSC is critical and enables the maintenance of the CSC subpopulation within a tumor and SOX2 has shown to mediate this in breast, gastric, ovarian, prostate cancer, glioma, osteosarcoma, lung adenocarcinoma and NSCLC [15,42,54,59,65,66,77,82,83]. Studies in gastric cancer using siRNA-mediated SOX2 knockdown, found reduced spheroid colony formation and increased apoptosis within sphere cells, highlighting the importance of SOX2 in self-renewal capacity [83]. In prostate cancer stem cells, the activation of EGFR signaling increased *SOX2* expression and the self-renewal capacity [59]. Singh and colleagues showed similar results in NSCLC, when siRNA-mediated SOX2 knockdown led to a 2.5-fold reduction in sphere formation [65]. Moreover, EGFR/Src/Akt signaling influenced SOX2 protein expression since during EGFR or SRC inhibition using gefitinib or BIBW, respectively, levels of SOX2 were considerably decreased [65]. Taken together, SOX2 mediates self-renewal of CSCs through EGFR signaling in at least two cancer types and is a major mediator of self-renewal in several cancers through mechanisms that remain unclear.

Recent CSC studies have concentrated on *SOX2* and its mechanisms in cancer stem cell maintenance and regulation. Santini and colleagues investigated SOX2 in melanoma initiating cells and Hedgehog-GLI (HH-GLI) signaling [50]. They functionally showed that the ectopic expression of SOX2 *in vitro* caused enhanced self-renewal capacity in melanoma cells. Moreover, GLI1 and GLI2 downstream transcription factors of HH-GLI Signaling were able to bind to the proximal promoter of SOX2 in primary melanoma cells in chromatin immunoprecipitation (ChIP) studies and therefore SOX2 is regulated by HH signaling [50]. To a similar extent, Justilien and colleagues heavily investigated Hedgehog (Hh) acyltransferase (HHAT) in LSCC and found that not only are *PRKCI* and *SOX2* coamplified and cooperate in LSCC but also SOX2 becomes phosphorylated by Protein Kinase Cι (PKCι) [61]. Next, phosphorylated SOX2 is recruited and required for HHAT expression and therefore maintenance of the stem-cell phenotype. The authors provide evidence that the coamplifcation of both oncogenes is required to activate the PKCi-SOX2-HHAT signaling axis which impels the stem cell phenotype. Further work is being done on establishing PKCι inhibitors for LSCC treatment [61].

Lastly, a recent publication by Favaro and colleagues proved that SOX2 is required for *in vitro* CSC maintenance in a high-grade oligodendroglioma mouse model [43]. Oligodenrogliomas were generated in mice *via* the transduction of PDGF-B-IRES-GFP-encoding retrovirus within the brain at embryonic day 14.5. Additionally these embryos carried a homozygous SOX2^flox^ mutation, which allowed authors to excise SOX2 using lentiviral Cre recombinase virus. Wild-type oligodendroglioma cells from mentioned mouse model were transplanted into the brain of C57/Bl6 mice, which generated lethal tumors. However, when SOX2-deletion cells were transplanted into C57/Bl6 mice they remained tumor free [43]. This elegant study proved the obligatory function of SOX2 in oligodendroglioma tumor initiation. The requirement of SOX2 in oligodendroglioma suggested possible therapeutic intervention.

### Correlations between SOX2 and clinical outcome: current research

SOX2 has proven its functional role in various aspects of cancer biology. Research on SOX2 has also investigated its importance in the clinic in respect to disease prognosis, relapse, therapy resistance, comprehensive summary in Table 2. The ability to improve reliability of diagnosis or prognosis of a cancer patient can have immense impact on survival and better understanding of the disease. For example, 162 esophageal squamous cancer patients were analyzed for SOX2 and OCT3/4 expression and high expression of both markers was associated with higher histological grade or TNM stage (p < 0.001 for both factors), demonstrating their link to dedifferentiation in these tumors (Table 2) [84]. Furthermore, a significant correlation between high SOX2 levels and decreasing patient survival was shown (p < 0.001) [84]. Recently, Forghanifard and colleagues revealed that stemness state regulators SALL4 and SOX2 are overexpressed in 64 esophageal cancer samples and co-overexpression correlated with depth of tumor invasion and metastasis [85]. Again, contrasting results for SOX2’s role in the clinic were highlighted in lung cancer (including NSCLC and SCLC), where SOX2 was correlated to improved survival and better patient outcome [62,67,68,86-88]. These opposing outcomes in the clinic further underscore the differing role of SOX2 in varying cancer types.

**Table 2:** Clinical relevance of SOX2

**Cancer type**	**Function in the clinic**	**References**
Breast	Promotes Tamoxifen resistance *via* WNT signaling activation in CSCs	[21,27,89]
	ZF-based ATF therapy effective for downregulation of SOX2	
	Expression correlates with TNM stage and histological grade	
Colorectal	Prognostic marker for metastasis	[29,32]
	Associated with poor patient prognosis	
	Associated with distant metastasis and lymph-node metastasis	
Esophageal	Co-expression with OCT3/4 significantly associated with higher histological stage	[84,85,90]
	Co-expression with OCT3/4 correlated to poorer survival	
	Co-expression with SALL4 correlates to depth of tumor invasion and metastasis	
	CD44 and SOX2 is correlated to poor survival	
Gastric	Associated with poor prognosis	[38,41]
	SOX2 methylation correlates to significantly shorter survival time	
	Predicts immunotherapy response	
Hepatocellular carcinoma	Expression correlates with metastasis	[47]
	Expression correlates with low survival rate	
Lung, NSCLC, Squamous cell lung cancer	Associated with better survival independent of histological subtype	[62,67,68,86-88]
	Expression is a positive prognostic marker	
	SOX2 amplification and upregulation are frequent events linked to favorable prognosis	
	Important tumor-associated antigen	
	Associates SOX2-positive T-cells to patient response to immunotherapy	
	Increases resistance to EGFR inhibitors	
Melanoma	Novel biomarker for normal skin subpopulation responsible for tumorigenesis	[52,53]
	SOX2 and Nestin differentiate between nevi and melanoma metastasis	
	SOX2 and Nestin powerful diagnostic tools	
Ovarian	Expression directly proportional to higher degree of malignancy	[56,82]
	Responsible for CSC therapy resistance	
Rectal	Predicts poor distance recurrence for preoperative CRT patients	[91]
	Predicts poor prognosis for preoperative CRT patients	
Sinonasal	SOX2 amplification identifies carcinomas more likely to relapse	[71]

The role of SOX2 in the clinic has been studied and expression has been correlated to patient survival, prognosis and therapy. SOX2 has been shown to play different roles depending on cancer type and can predict both better and worse outcomes for patients. Moreover, SOX2 has proven its use as a prognostic marker in melanoma, colorectal, gastric and lung cancer.

### SOX2’s influence on therapy resistance

Drug resistance has been associated with expression of various pluripotency markers, including NANOG, OCT4 and SOX2, since these genes typically lead to a decrease in differentiation status [68]. In breast cancer, silencing SOX2 not only reduced the size of the CSC population but also restored tamoxifen sensitivity, suggesting tamoxifen resistance is primarily driven by SOX2 in breast CSCs [89]. Recently, Dogan and colleagues investigated SOX2 and EGFR inhibitors in lung adenocarcinoma cell lines [68]. When a SOX2 knockdown using shRNA was performed in HCC827 cells, decreased proliferation was observed along with increased sensitivity to erlotinib. Furthermore, upon the treatment with PI3K/AKT inhibitors, SOX2 expression decreased [68]. Therefore, targeting SOX2 in EGFR-mutant tumors may be therapeutically beneficial; however more direct targeting strategies need to be further developed.

### SOX2 and therapy options

Improving cancer therapy options in the clinic remains a priority. SOX2 has shown its potential to become a useful biomarker in the clinic for some cancer types, for both staging tumors and identification of the CSC subpopulations (Table 2). Utilizing SOX2 for cancer therapy may open a window to new therapeutic opportunities. SOX2 does not lend itself for direct therapeutic intervention due to its importance in transcriptional cellular function and targeting SOX2 may have dozens of unwanted complications. However, targeting signals upstream or downstream of SOX2 may prove beneficial in cancer therapy. For example, as mentioned above in NSCLC and prostate CSCs, SOX2 relies heavily on EGFR signaling for mediating self-renewal in CSCs. Currently, anticancer drugs are on the market targeting EGFR, such as gefitinib and erlotinib, and may be useful in inhibiting SOX2’s downstream self-renewal effects, however resistance to these therapies is nearly inevitable and needs to be overcome. Therefore, further targeting SOX2 in EGFR-mutant cancer with PI3K/AKT inhibitors, as shown above in lung adenocarcinoma cell lines, may yield better results [68].

Recently published studies have attempted to tackle SOX2 therapy and its implications. Favaro and colleagues, who performed the oligodenroglioma studies in mice, further examined SOX2 peptides for immunotherapy treatment in mice [43]. The C57BL/6 N mice were injected with wild-type oligodendroglioma cells from established mouse model and upon vaccination with SOX2 peptides, significant delay in tumor growth was observed [43]. Similarly, Polakova and colleagues developed an experimental DNA vaccine against SOX2 [92]. In this study, C57BL/6 and BALB/c mice were immunized with DNA vaccine and found a significant SOX2-specific activation of lymphocytes. However, when the antitumor effects were examined using TC-1/B7 (derived from lung cancer cell line TC-1) cells the DNA vaccination did not prevent tumor development even though it was able to significantly reduce tumor growth [92].

## Conclusion: SOX2 in the future

SOX2 has proven not only to be an essential embryonic, reprogramming and development-associated gene but has begun to leave its footprint in the field of oncology. SOX2 is intricately involved in many cancer-associated processes such as cell proliferation, evading cellular apoptosis and metastasis *via* interactions with EGFR signaling and several other oncogenic pathways and processes. Moreover, current and ongoing SOX2 CSC research has emphasized the importance of investigating early developmental genes, since they may be responsible for self-renewal of CSCs. Lastly, in clinical settings, SOX2 has shown a heavy influence on patient survival and prognosis. In summary, SOX2 function in cancer has been accentuated in numerous cancer types in and out of the clinic and investigating SOX2’s oncogenic course is important for future prognosis, survival of cancer patients and possible therapeutic interventions.

## Abbreviations

CCND3: CyclinD3; CDH1: E-Cadherin; ChIP: Chromatin immunoprecipitation; CRT: Chemoradiotherapy; CSCs: Cancer stem cells; CTNNB1: β-Catenin; EGFR: Epidermal growth factor receptor; EMT: Epithelial-mesenchymal-transition; GBM: Glioblastoma multiforme; HH: Hedgehog; LSCC: Lung squamous cell carcinoma; mTOR: Mammalian target of rapamycin; MET: Mesenchymal-epithelial-transition; MMP: Matrix metalloproteinase; NOD/SCID: Non-obese diabetic-severe combined immunodeficiency; NSCLC: Non-small-cell lung cancer; PI3K: Phosphatidylinositol 3-kinase; PCSC: Prostate cancer stem cell; SCC: Squamous cell carcinoma; SCLC: Small-cell lung cancer; ZF-based ATF: Zinc finger-based artificial transcription factor.

## Competing interests

The authors declare that they have no commercial or other competing interests to disclose.

## Authors’ contributions

The author KW performed the research, writing, and revisions of the manuscript. Additionally, author KW created the figures and tables. The authors KW and JU contributed equally to the revisions of the manuscript and figures. Both authors read and approved the final manuscript.
